# Ulvophyte Green Algae *Caulerpa lentillifera*: Metabolites Profile and Antioxidant, Anticancer, Anti-Obesity, and In Vitro Cytotoxicity Properties

**DOI:** 10.3390/molecules28031365

**Published:** 2023-01-31

**Authors:** Fahrul Nurkolis, Nurpudji Astuti Taslim, Faqrizal Ria Qhabibi, Sojin Kang, Myunghan Moon, Jinwon Choi, Min Choi, Moon Nyeo Park, Nelly Mayulu, Bonglee Kim

**Affiliations:** 1 Department of Biological Sciences, State Islamic University of Sunan Kalijaga (UIN Sunan Kalijaga), Yogyakarta 55281, Indonesia; 2 Department of Clinical Nutrition, Faculty of Medicine, Hasanuddin University, Makassar 90245, Indonesia; 3 Medical School Department, Faculty of Medicine, Brawijaya University, Malang 65145, Indonesia; 4 Department of Pathology, College of Korean Medicine, Kyung Hee University, Seoul 02447, Republic of Korea; 5 Korean Medicine-Based Drug Repositioning Cancer Research Center, College of Korean Medicine, Kyung Hee University, Seoul 02447, Republic of Korea; 6 Nutrition and Food, Faculty of Medicine, Sam Ratulangi University, Manado 95115, Indonesia

**Keywords:** anti-obesity, anticancer, antioxidant activity, *Caulerpa lentillifera*, cytotoxicity, functional food, green algae, secondary metabolites

## Abstract

Marine algae have excellent bioresource properties with potential nutritional and bioactive therapeutic benefits, but studies regarding *Caulerpa lentillifera* are limited. This study aims to explore the metabolites profile and the antioxidant, anticancer, anti-obesity, and in vitro cytotoxicity properties of fractionated ethanolic extract of *C. lentillifera* using two maceration and soxhlet extraction methods. Dried simplicia of *C. lentillifera* was mashed and extracted in ethanol solvent, concentrated and evaporated, then sequentially partitioned with equal volumes of ethyl acetate and *n*-Hexane. Six samples were used in this study, consisting of ME (Maceration—Ethanol), MEA (Maceration—Ethyl Acetate), MH (Maceration—*n*-Hexane), SE (Soxhletation—Ethanol), SEA (Soxhletation—Ethyl Acetate), and SH (Soxhletation—*n*-Hexane). Non-targeted metabolomic profiling was determined using LC-HRMS, while antioxidant, anti-obesity, and anticancer cytotoxicity were determined using DPPH and ABTS, lipase inhibition, and MTT assay, respectively. This study demonstrates that *C. lentillifera* has several functional metabolites, antioxidant capacity (EC_50_ MH is very close to EC_50_ of Trolox), as well as anti-obesity properties (EC_50_ MH < EC_50_ orlistat, an inhibitor of lipid hydrolyzing enzymes), which are useful as precursors for new therapeutic approaches in improving obesity-related diseases. More interestingly, ME, MH, and SE are novel bioresource agents for anticancer drugs, especially for hepatoma, breast, colorectal, and leukemia cancers. Finally, *C. lentillifera* can be a nutraceutical with great therapeutic benefits.

## 1. Introduction

The algae group belonging to the genus Caulerpa, especially *Caulerpa racemosa* and *Caulerpa lentillifera*, are types of ulvophyte green algae that are widely consumed in the Pacific and Southeast Asian regions. Asia accounted for 97.4% of global seaweed production in 2019 (99.1% of cultivation), with seven of the ten largest-producing countries located in East or Southeast Asia [1,2]. Until now, the main aquaculture production that has high economic value is *Caulerpa lentillifera*, which is also traded internationally in Pacific countries [3,4]. Although the consumption of macroalgae is not as common in Europe as in Asia, microalgae have gained popularity due to their physiologically active components, earning them the nickname “new superfood” [5]. Groups of algae in the genus Caulerpa are recognized as functional food sources due to the presence of secondary metabolites. These algae are rich in carbohydrates, proteins, unsaturated fatty acids, and vitamin complexes, and have a much higher mineral content when compared to terrestrial vegetables [6,7,8,9]. However, the aquaculture-production potential of many varieties of Caulerpa sea grapes is rarely studied and has never been used in a high-density commercial-scale system [3]. The global harvest of macroalgae in 2013 was estimated at USD 6.7 billion, with more than 95% produced in sea-algae-farming countries [10]. About two-thirds of Indonesia’s territory is in the form of the sea, and it is famous as one of the world’s mega-diversity areas, with more than 555 macroalgae species having been identified in its ocean areas. In addition, most of Indonesia’s islands are located within the Coral Triangle, which has been identified as an area with a very high diversity of Caulerpa [11,12,13].

*Caulerpa lentillifera* originated in tropical areas around India and the Pacific Ocean, such as Indonesia, but today it can also be found in the Korean Peninsula due to changes in global temperatures. The genus Caulerpa is famous as an edible species that has a high nutritional content, such as minerals, dietary fibers, vitamin A, vitamin C, and several essential unsaturated fatty acids [14,15]. The genus Caulerpa has also been used to treat a wide variety of diseases. For instance, anti-inflammatory, antioxidant, antimicrobial, lipid-lowering, and anticancer properties, as well as cardiovascular protection, renal protection, hepatoprotection, and neuroprotection, are just a few of the previously described medicinal activities of seaweed [16]. Unlike terrestrial plants, marine algae have not been widely used as an alternative medicine or adjuvant to medicines. However, technological advances and innovative engineering have allowed organisms in the marine environment to be used for scientific experiments in the last 50 years [17].

Interestingly, natural populations of *C. lentillifera* have varying nutritional and biochemical properties due to environmental factors such as predation, sedimentation, salinity, temperature, pollution, and nutrients; thus, different geographical growing fields can contribute to varying levels of nutrients and secondary metabolites [11]. Despite its abundance, research on Indonesian *C. lentillifera*’s bioactive molecules profile and their direct biological activities is still limited. Due to the uniqueness of each alga and its particular characteristics, exploration of the multifunctional properties of these algae has become urgent. Therefore, in this study, two different extraction methods (maceration and soxhletation) were used, with each extraction using three solvents each having different polarities, namely *n*-Hexane (non-polar), ethyl acetate (semi-polar), and ethanol (polar), in order to elucidate different compounds on the basis of the degree of polarity of the compounds. To date, there have been no studies profiling and exploring the compounds or metabolites of Indonesian *C. lentillifera*, especially those related to bioactivity in various kinds of non-communicable diseases, such as cancer and obesity-related diseases. Therefore, this study urgently aimed to profile the metabolites and the antioxidant, anticancer, anti-obesity, and cytotoxicity properties of Indonesian *C. lentillifera*.

## 2. Results

### 2.1. Metabolites Profile of Caulerpa lentillifera via HPLC-ESI-HRMS/MS Analysis

Using two different ethanol extraction methods (maceration and soxhletation), as well as solvent types sequentially partitioned with equal volumes of *n*-Hexane and ethyl acetate, *Caulerpa lentillifera* samples with different metabolites profiles were successfully obtained and analyzed using non-targeted metabolomic profiling HPLC-ESI-HRMS/MS analysis (Table 1). The data shown in Table 1 are based on matches to the library of mzCloud Best Match (>99%) and are sorted according to their abundance.

The highest abundance of compounds was obtained from *C. lentillifera* ethanolic macerated-extract fraction (ME), with as many as eight metabolites. Furthermore, six compounds were obtained from MEA, five metabolites from MH, seven compounds from SE, six from SEA, and five from SH. The abundance and type of compounds obtained in this study depended on the extraction method and solvent used.

### 2.2. Radical Scavenging Activity (Antioxidant Properties) of Caulerpa lentillifera

**Figure 1:**
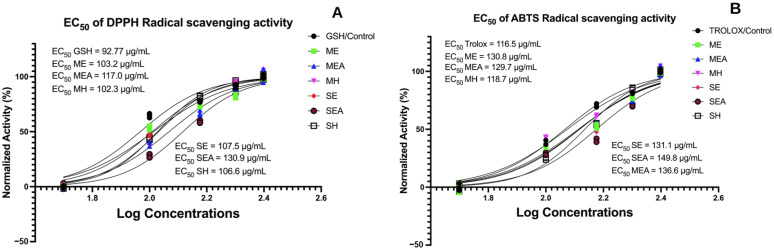
Antioxidant capacity of *C. lentillifera*. (**A**) The half-maximal effective concentration (EC_50_) of DPPH radical scavenging activity. (**B**) The half-maximal effective concentration (EC_50_) of ABTS radical scavenging activity. **ME**: Maceration—Ethanol; **MEA**: Maceration—Ethyl Acetate; **MH**: Maceration—*n*-Hexane; **SE**: Soxhletation—Ethanol; **SEA**: Soxhletation—Ethyl Acetate; **SH**: Soxhletation—*n*-Hexane.

The scavenging activity of free radicals determines antioxidant properties due to its reduction–oxidation (redox) properties. It is important to see the potential of *Caulerpa lentillifera* as a functional food candidate in reducing various diseases, including cancer and obesity, by improving oxidative stress. The antioxidant potential in this study was evaluated using DPPH and ABTS, and the antioxidant activity observed depended on the concentration dose used (Figure 1).

In the DPPH, it was seen that the antioxidant potential of *C. lentillifera* in ME, MH, SE, and SH had EC_50_ values of 103.2 μg/mL, 102.3 μg/mL, 107.5 μg/mL, and 106.6 μg/mL, respectively, compared to glutathione as a positive control (GSH), which showed an EC_50_ value of 92.77 μg/mL (Figure 1A). In the ABTS radical scavenging assay, it appears that *C. lentillifera* in MH has the potential to be an antioxidant compared to Trolox as a positive control (EC_50_ MH is very close to EC_50_ Trolox; Figure 1B).

### 2.3. The Anti-obesity Potential of Caulerpa lentillifera via Lipase Inhibitory Activity

**Figure 2:**
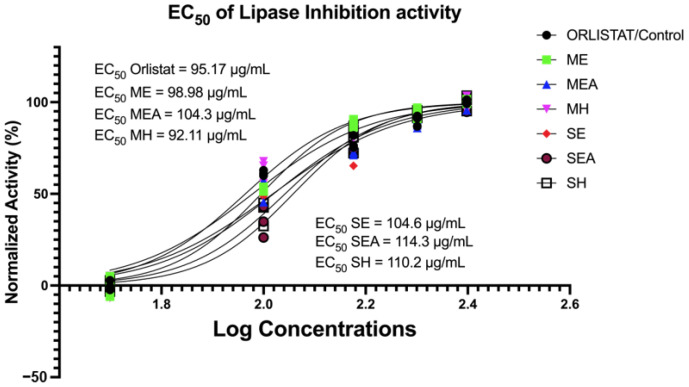
The half-maximal effective concentration (EC_50_) of lipase inhibition activity. **ME**: Maceration—Ethanol; **MEA**: Maceration—Ethyl Acetate; **MH**: Maceration—*n*-Hexane; **SE**: Soxhletation—Ethanol; **SEA**: Soxhletation—Ethyl Acetate; **SH**: Soxhletation—*n*-Hexane.

The inhibitory activity of lipase determines anti-obesity properties due to its inhibitory properties to lipid (lipase) hydrolyzing enzymes, and it is important to see the potential of *Caulerpa lentillifera* as a functional food candidate in reducing various diseases, including obesity. Anti-obesity potential in this study was evaluated using in vitro lipase inhibitory activity in a dose-dependent manner (Figure 2).

It was shown that *C. lentillifera*, especially in MH, shows more potent anti-obesity activity than the standard drug orlistat, which was a positive control (EC_50_ MH < EC_50_ orlistat). This is in line with the results in Figure 1B, which highlight MH having the highest antioxidant properties compared to other groups. Furthermore, the anti-obesity potential of *C. lentillifera* in ME, MEA, SE, SH, and SEA exhibited EC_50_ values of 98.98 μg/mL, 104.3 μg/mL, 104.6 μg/mL, 110.2 μg/mL, and 114.3 μg/mL, respectively, approaching the EC_50_ values of orlistat (95.17 μg/mL; Figure 2).

### 2.4. Cytotoxicity Properties of C. lentillifera

The LC_50_ value of *C. lentillifera* in a cytotoxicity test in normal cell lines is presented in Table 2. The LC_50_ sequence of samples that were lowest or showing the highest cytotoxicity to the lowest cytotoxicity is SE, ME, SEA, MH, MEA, and SH at 24 incubation hours; and MEA, SE, ME, MH, SEA, and SH at 48 h (Table 2).

In general, the LC_50_ value of *C. lentillifera* > 500 μg/mL, and this result suggested that *C. lentillifera* (ME, MEA, MH, SE, SEA, and SH) as an antioxidant with anti-obesity agents in the observed EC_50_ was safe. Furthermore, in terms of cytotoxicity, it was observed that *C. lentillifera* was safe to be potentially developed into various products, especially future functional food products.

### 2.5. Antiproliferative Activity of Caulerpa lentillifera

When considering the anticancer potential of *C. lentillifera* via proliferation inhibition of ethanolic extract and its fractions, ME, MH, and SE showed some potential against various observed cancer cells (Table 3). For example, ME (IC_50_: 160.80 μg/mL) and MH (IC_50_: 100.50 μg/mL) showed stronger activity on the KAIMRC1 cell line, while MH with IC_50_: 283.00 μg/mL showed high activity on the HEP G2 cell line, thus indicating its potential as an anticancer agent in hepatoma. Similarly, ME (IC_50_: 170.10 μg/mL), MH (IC_50_: 184.00 μg/mL), and SE (IC_50_: 160.50 μg/mL) showed high activity on the colorectal HCT-8 cell line. Furthermore, ME (IC_50_: 320.50 μg/mL), MH (IC_50_: 280.50 μg/mL), and SE (IC_50_: 104.10 μg/mL) also showed high activity on cell line MDA-MB-231, followed by ME (IC_50_: 100.90 μg/mL) and MH (IC_50_: 350.20 μg/mL), which showed high activity on the MCF-7 cell line. Even MH showed high activity against KG-1a, with IC_50_ of 340.50 μg/mL, thus showing its potential as an anticancer agent in breast cancer. Interestingly, ME with an IC_50_ of 315.50 μg/mL also showed high activity on the HL-60 leukemia cell line. As in the normal cell cytotoxicity shown in Table 2, IC_50_ on the normal/control cell lines (monocular blood/PBMC and epithelial) was more than 500 μg/mL, which indicated that it was safe.

## 3. Discussion

This study intended to explore the metabolites profile and the antioxidant, anti-obesity, anticancer, and in vitro cytotoxicity of fractionated ethanol extract of *C. lentillifera*, ulvophyte green algae. The Mediterranean diet that has been recognized worldwide is associated with a reduced risk of cancer, heart disease, obesity, stroke, and other non-communicable diseases [18,19]. This lower risk is universally ascribed to a high intake of vegetables and fruits and, thus, high consumption of phytochemicals, including marine products. This study explored *Caulerpa lentillifera* using two different ethanol extraction methods (maceration and soxhlet), and the highest abundance of compounds was obtained from *C. lentillifera* ethanolic macerated-extract fraction (ME) (Table 1). The main compound in the most abundant ME was 3-[3-(beta-d-Glucopyranosyloxy)-2-hydroxyphenyl]propanoic acid (C_15_H_20_O_9_), which in previous studies showed great activity as an anti-obesity agent and molecular antidiabetic [20]. In fact, in this study, many fatty acids and their derivatives were also found, such as α-linolenic acid (in ME), oleamide (in MH), α-eleostearic acid and palmitoleic acid (in SE), and γ-linolenic acid ethyl ester (in SH) (Table 1). Evidence from systematic review and meta-analysis suggests that consumption of fatty acids can lower the risk of cardiometabolic syndrome and restore imbalances in lipid and glucose metabolism, and thus may contribute to the prevention and treatment of cardiovascular diseases, including obesity [21]. Ouabain (in MEA) and cafestol (in SEA) were also observed in *C. lentillifera*. Cafestol can suppress the rapid growth and migration of cancer cells [22], while ouabain is mainly used in the treatment of congestive heart failure and arrhythmias [23], and even as an anticancer agent in melanoma [24].

Currently, there is great interest in the development of new nutraceuticals of natural origin, in particular marine bioresources for use in pharmaceuticals and/or as potential dietary supplements. This demonstrates the importance of *C. lentillifera*’s role in the development of future drugs and/or dietary supplements. It is even more interesting that its cytotoxicity properties are considered safe since the lethal concentration (LC_50_) is >500 μg/mL, implying fewer cytotoxic properties in normal cells (Table 2), based on National Cancer Institute criteria [25]. This is in line with similar studies on the cytotoxicity of subcritical water extraction of genus Caulerpa [26], which did not show significant cytotoxicity activity, and further findings from a review study by Aroyehun et al., (2020), which stated that the toxicity was minimal [27]. Therefore, further development of *C. lentillifera* products will be of interest to many scientists. In line with the finding by Syakilla et al. [5] that algae is a “new superfood”, recent research has also shown the potential of *C. lentillifera* as a promising agent against SARS-CoV-2 [28].

One of the most significant and most promising approaches to treatment for obesity and cancer is to fight cellular pro-oxidant states by suppressing the production of reactive oxygen species (ROS) and subsequent oxidative stress [29,30]. Natural antioxidants can present a viable therapeutic target in helping to correct the negative effects of obesity, cancer, and oxidative stress [31,32]. You et al. [33] have studied isolated dietary fiber from *C. lentillifera* for the first time to identify its potential anti-obesity activity. However, the study by You et al. [33] has not shown potential anti-obesity effects through the lipase inhibition pathway. This study demonstrates that *C. lentillifera* can be an antioxidant agent with anti-obesity effects since it has an antioxidant capacity (EC_50_ MH is very close to EC_50_ Trolox/control positive) followed by anti-obesity properties via lipase inhibition (EC_50_ MH < EC_50_ orlistat/control positive)—a useful precursor to new therapeutic approaches in improving obesity-related diseases. Lipase inhibitors are clinically used in obese individuals to improve lipid metabolism [34]. Lipase inhibitors, by inhibiting the absorption of fatty acids, reduce the buildup of free fatty acids, which also lowers LDL levels in serums [35]. Furthermore, cumulative evidence suggests that antioxidants can modulate target and dynamic cellular processes to correct redox imbalances in obesity and cancer [36,37]. Therefore, this study has succeeded in uncovering antioxidants and novel lipase inhibitors that have the potential to improve obesity, cancer, and related comorbidities. Furthermore, in addition to its potential as an antioxidant and anti-obesity agent, *C. lentillifera* has anticancer capacity, in particular in ME, MH, and SE as novel bioresource agents for anticancer drugs, especially for human hepatoma, breast, colorectal, and leukemia cancers. As regards anticancer activity, *C. lentillifera* has more broadly demonstrated its anticancer properties than other species, for example *Caulerpa racemosa*, which has only shown its best potential as a drug candidate for breast cancer [17].

Therefore, this original study is dedicated to providing a view of the latest findings on the best extraction techniques for the recovery of bioactive constituents and the chemical characterization of phytoextracts with valuable biological activity from *C. lentillifera*. Furthermore, it is hoped that further extensive antioxidant assays can complement the result of this publication, for example by exploring the use of the ESR spectrometer method in determining antioxidant activity. Moreover, the research that is being planned involves in vivo and in vitro bioassays and even human clinical trials to further evidence the biological activity of purified compounds and complex extracts and/or combined extracts derived from different matrices of *C. lentillifera*. In addition, it is hoped that, in the future, the presentation of data on the compounds examined in this study can be reproduced by other researchers using a computational approach, such as in silico (molecular docking and dynamics simulations).

## 4. Materials and Methods

### 4.1. Preparation of Caulerpa lentillifera

**Figure 3:**
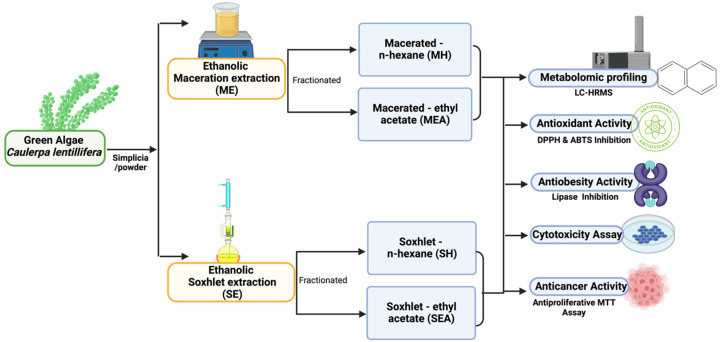
Methodical schematic of *Caulerpa lentillifera* study. **ME**: Maceration—Ethanol; **MEA**: Maceration—Ethyl Acetate; **MH**: Maceration—*n*-Hexane; **SE**: Soxhletation—Ethanol; **SEA**: Soxhletation—Ethyl Acetate; **SH**: Soxhletation—*n*-Hexane.

Fresh green algae (*Caulerpa lentillifera*) or sea grapes were collected from the Caulerpa cultivation pond in Jepara Regency, Central Java Province, Indonesia (6.5868° S latitude, 110.6444° E longitude). The owner of the *C. lentillifera* pond and the local authorities approved the *Caulerpa lentillifera* collection sample. Botanical identification and authentication were confirmed at the Integrated Laboratory of the State Islamic University of Sunan Kalijaga (UIN Sunan Kalijaga), Yogyakarta-55281, Indonesia, in accordance with the National Center for Biotechnology Information (NCBI) Taxonomy ID: NCBI: txid148947 and Integrated Taxonomic Information System—Report ID 6973. The researchers (authors) state and confirm that all methods used in this study comply with relevant in vitro and algae research guidelines and regulations. Additionally, the entire body of *C. lentillifera* to be used was thoroughly washed so that any dirt adhering to it was removed and the plant became clean. *C. lentillifera* that had been washed, drained, and sun-dried for two to three days were then dried in an oven (IN55 Memmert Incubator; Schwabach, Germany) at 60 degrees Celsius. The dried *C. lentillifera* was then chopped into small pieces and pulverized in a blender to produce *C. lentillifera* simplicia powder. The dried simplicia was pulverized and then extracted using maceration and the soxhlet method (Figure 3).

### 4.2. Caulerpa lentillifera Extraction

The dried simplicia was extracted using either the hot or the cold method [11]. Maceration represents the cold way, while soxhlet represents the hot way.

#### 4.2.1. Maceration Extraction Method (Cold Extraction)

One kilogram of sea grape powder (*C. lentillifera*) was placed in a dark bottle, to which was added 2 L of 96% ethanol solvent in a ratio of 1:2. The simplicia was soaked for three periods of 24 h. Every 1 × 24 h, the obtained filtrate was stirred occasionally and filtered with Whatman 41 paper, and the residue was re-macerated in a new 96% ethanol solvent. The extraction results were then concentrated using a rotary evaporator under low pressure (100 millibars) for 90 min, evaporated in a 40 °C oven, and sequentially partitioned with equal volumes of ethyl acetate and *n*-Hexane to yield a thick extract of *C. lentillifera* (Figure 3).

#### 4.2.2. Soxhlet Extraction Method (Hot Extraction)

A soxhlet device was installed, then 50 g of sea grape powder (*C. lentillifera*) wrapped in filter paper was placed in a soxhlet tube (thimble). A total of 250 mL of a 96% ethanol solvent was added and divided into two parts: 150 mL was placed in the soxhlet flask (round base flask), and 100 mL was placed in the soxhlet tube to wet the simplicia. The proportion of simplicia to solvent was 1 to 5. The soxhletation procedure was performed at between 70 and 80 degrees Celsius. The extraction time was up to three cycles, and the extraction results were partitioned sequentially with equal volumes of ethyl acetate and *n*-Hexane (Figure 3).

### 4.3. Metabolomic Profiling Analysis

In accordance with Permatasari et al., (2022) [20], the untargeted metabolomics profiling test (to view the compounds profile) on the samples (ME, MEA, MH, SE, SEA, and SH; Figure 1) was performed by Laboratorium Sentral Ilmu Hayati (LSIH/Central Laboratory of Life Sciences, Brawijaya University, Malang-65145, Indonesia; ISO 9001:2008 and ISO 17025:2005), test number 041-LSIH-UB-LK. Then, 50 µL of samples were centrifuged for 2 min at 6000 rpm after being vortexed 30 times with ethanol (96%) in a volume of 2000 rpm. Before analysis, 0.22 m syringe filters were used to filter supernatants. The LC-HRMS system consisted of a Thermo Scientific Dionex (Waltham, MA, USA) Ultimate 3000 RSLC Nano High-Performance Liquid Chromatography (HPLC) instrument and a micro flow meter. The analytical column was a 1 mm × 1.9 m Hypersil GOLD aQ 50 column maintained at 30 °C. Solvents A and B contained 0.1% formic acid dissolved in water and 0.1% formic acid dissolved in acetonitrile, respectively. Separation was effected using a linear gradient at a flow rate of 40 L/min for 30 min. The Thermo Scientific Q Exact had a full-scan resolution of 70,000, a data-dependent MS2 resolution of 17,500, and a 30-min operation period in both positive and negative modes.

### 4.4. Antioxidant Activity by ABTS and DPPH Radical Scavenging Activity Assay (ABTS and DPPH Inhibition) (%)

The scavenging of 2,2′-Azino-bis(3-ethylbenzothiazoline-6-sulfonic acid) or diammonium salt radical cation (ABTS+; Sigma-Aldrich; Saint Louis, MO, USA) was based on the procedure outlined by Permatasari [38]. Potassium persulfate (2.4 mM) and ABTS (7 mM) were mixed in a 1:1 ratio, shielded from light with aluminum foil, and allowed to react for 14 h at 22 degrees Celsius. To obtain a working solution with an absorbance of 0.706 at 734 nm, the mixture was further diluted (e.g., 1 mL of the stock solution plus 60 mL of ethanol). For each test, a fresh working solution was formulated. The samples’ (ME, MEA, MH, SE, SEA, and SH) extracts (50, 100, 150, 200, and 250 µg/mL) were diluted with ABTS working solution (1 mL), and the absorbance at 734 nm was measured after 7 min. As a positive control, the known antioxidant molecule Trolox was utilized. The antioxidant activity in the (2,2-diphenyl-1-picrylhydrazyl radical scavenging activity) (DPPH) test was assayed in accordance with Permatasari’s procedure [38]. DPPH solution was prepared by dissolving 24 mg of DPPH in 100 mL of methanol. In the testing vial, a concentration of 50, 100, 150, 200, and 250 μg/mL of samples (ME, MEA, MH, SE, SEA, and SH) was added to the DPPH reagent (3 mL). The DPPH-samples mixture was then incubated at room temperature for 30 min. Change in the concentration of DPPH was observed based on 517 nm absorbance. Glutathione (GSH; Sigma-Aldrich, 354102) was used as a positive control.

To ensure the validity of the data results (ABTS and DPPH tests), each sample went through the above procedures thrice (triplicates test; *n* = 3).

Inhibition of DPPH and ABTS was expressed as a percentage and determined according to the formula below:

(1) $$\mathrm{Inhibition}\;\mathrm{Activity}\;\left(\%\right)=\frac{A0-A1}{A0}\times 100\%$$

A0 = absorbance of blank; A1 = absorbance of standard or sample.

The half-maximal effective concentration ratio (EC_50_), which is defined as the sample concentration that results in a 50% decrease in the initial radical concentration, was used to express the radical scavenging ability of the samples and Trolox for ABTS, and the samples and glutathione for the DPPH assay.

### 4.5. Cytotoxicity Evaluation of Caulerpa lentillifera Using MTT Assay

A human Caucasian skin fibroblast cell line (Normal cell; Bud-8) was used to assess the cell viability [39]. The rate of Bud-8 cell-line proliferation following treatment was determined using a 3-(4,5-dimethylthiazol-2-yl)-2,5-diphenyl tetrazolium bromide (MTT) assay. Mitochondrial dehydrogenase can convert MTT to Formazan, a purple and water-insoluble compound, based on the viability of the cell. Cells were maintained in Dulbecco’s Modified Eagle’s Medium (DMEM), supplemented with 10% fetal bovine serum (FBS) and 1 × Penicillamine-Streptomycin-Neomycin (PSN). Then, 100 microliters of cells (4 × 10^4^ cells/mL) were seeded in a 96-well plate and incubated for 24 h at 37 °C with 5% carbon dioxide. After 24 h of incubation, 100 µL of samples containing 100, 200, 300, 400, and 500 µg/mL was added to 100 µL of cells (ME, MEA, MH, SE, SEA, and SH). The plate was incubated for 24 and 48 h at 37 °C with 5% CO_2_. After incubation, the morphology of the cells was examined under a microscope. Sigma supplied 20 microliters of a 5 mg/mL MTT solution per well plate. Following an additional two to four hours of incubation, the medium was drained from the plate. Crystals of formazan were dissolved in 100 µL of dimethyl sulfoxide (DMSO; Sigma). The percentage of viable cells and LC_50_ cells was estimated using the following formula:

(2) $$\%\;\mathrm{Cell}\;\mathrm{Viability}=\frac{A1}{A0}\;\times \;100\%$$

where A0 is absorbance control in cells given 1% DMSO and A1 is absorbance control in cells given the test sample.

Lethal concentration (LC_50_) is the lowest concentration of samples that inhibits 50% of cells. In general, a low LC_50_ value indicates high toxicity. Extracts with high LC_50_ are preferred for use due to their low toxicity effect on host cells [39].

### 4.6. Anticancer Evaluation of Caulerpa lentillifera via Antiproliferative Activity

The protocol study [17], in which antiproliferative experiments were conducted, was granted institutional review board (IRB) approval. The American Type Culture Collection (ATCC) supplied cell lines for colorectal cancer (HCT-8), human breast cancer (MCF-7), hepatic cancer (Hep G2), leukemia (lymphoblastic: K-562; acute myeloid leukemia: HL-60; erythroleukemia: KG-1a), breast epithelial cancer (MDA-MB-231), and normal breast epithelial cancer (MCF-10A). Primary blood mononuclear cells (PBMC) and the KAIMRC 1 cell line were cultured in the authors’ laboratory. Purchased from Sigma-Aldrich^®^, the positive control was mitoxantrone (C_22_H_28_N_4_O_6_; CID 4212).

In accordance with the manufacturer’s instructions, the CellTiter-Glo assay (Promega; Madison, WI, USA) was utilized to determine the effect of algal fractions on the proliferation of non-adherent cells. An EnVision (London, UK) plate reader was utilized to quantify luminescence, which was then normalized to average DMSO controls and expressed as a percentage. Each portion of the extracts had a concentration ranging from 0 to 250 µg/mL, and the cells were seeded in 96-well plates containing a growth medium. After incubating the cells at 37 °C for 24 h, CellTiter-Glo reagent was added to each well and stirred for 2 min. Then, luminescence was measured using the EnVision plate reader (Perkin Elmer; Buckinghamshire, UK). Each compound’s IC_50_ values (µg/mL), or half-maximal inhibitory concentrations, were calculated [17].

The MTT test was conducted using the aforementioned method. To assess the impact of the algal fractions on the proliferation of adherent cells, the MTT reagent (Sigma) was used in place of the CellTiter-Glo reagent. Utilizing the Spectra Max spectrophotometer from Invitrogen, absorbance was measured. It was then adjusted to average DMSO controls and given as a percentage [40].

### 4.7. In Vitro Anti-Obesity via Lipase Inhibition Assay (%)

Initially, crude pig-pancreatic lipase (PPL, 1 mg/mL) was dissolved in phosphate buffer (50 mM, pH 7) and then centrifuged at 12,000× *g* to remove insoluble components. To create an enzyme stock (0.1 mg/mL), the supernatant was diluted 10-fold with buffer. Prior research was used to evaluate the lipase inhibition potential [13]. A transparent 96-well microplate containing 100 µL of samples (ME, MEA, MH, SE, SEA, and SH) was combined with 20 µL of p-nitrophenyl butyrate (pNPB,10 mM in buffer) and incubated for 10 min at 37 °C. The outcome was compared to the reference drug orlistat, a well-known PPL inhibitor. At 405 nm, measurements were taken using a microplate reader. The unit of activity was calculated using the yield from the reaction rate of 1 mol of p-nitrophenol per minute at 37 °C. To measure the lipase inhibition activity, PPL activity was reduced in the test mixture by a specific amount. To ensure the validity of the study results, each sample was verified three times (in triplicate). The inhibitory data were obtained using the equation below.

(3) $$\mathrm{Inhibition}\;\mathrm{of}\;\mathrm{Lipase}\;\mathrm{Activity}\;\left(\%\right)=100-\frac{B-\mathrm{Bc}}{A-\mathrm{Ac}}\times 100\%$$

A = activity without inhibitor; Ac—negative control without inhibitor; B—activity with inhibitor; Bc—negative control with inhibitor.

### 4.8. Management and Analysis of Data

For the metabolomic profiling study, compound data were sorted according to the Advanced Mass Spectral Database (*M*/*Z* cloud; https://www.mzcloud.org, accessed on 23 February 2022) best match criteria with a match rate of >99.0%. The statistical analysis was conducted using the MacBook version of GraphPad Prism 9 Software. Each EC_50_ and LC_50_ data set was generated utilizing nonlinear regression formulas to statistically evaluate data from the in vitro experiments, including antioxidant inhibition of ABTS, DPPH, cytotoxicity, and lipase inhibitory activities conducted three times. In addition, the calculated mean and standard deviation were displayed for all data.

## 5. Conclusions

On the basis of this study, and supported by some scientific evidence, *Caulerpa lentillifera* is an ulvophyte green alga that has several functional metabolites, antioxidant capacity, as well as anti-obesity properties, which are useful as precursors for new therapeutic approaches in improving obesity-related diseases. More interestingly, ME, MH, and SE are novel bioresource agents for anticancer agents, especially for hepatoma, breast, colorectal, and even leukemia cancers. Therefore, *C. lentillifera* has potential to be a functional food with strong therapeutic benefits and to be developed as a promising drug candidate in the future. However, in vivo trials and a clinical trial, which the authors are planning for the future, are urgently needed to confirm and validate these mechanisms.

## 6. Patents

The preparation method and formulation of *C. lentillifera* extract resulting from the work reported in this study have been registered as a patent in Indonesia with number P00202300540.

## Figures and Tables

**Table 1:** Metabolites profile observed in *C. lentillifera* from HPLC-ESI-HRMS/MS analysis.

Sample	Compounds	Molecular Formula	Retention Time (Min)	Area Max (Peak Area)	Observed MW HR-ESIMS *m*/*z*
**ME**	3-[3-(beta-d-Glucopyranosyloxy)-2-hydroxyphenyl]propanoic acid	C_15_H_20_O_9_	15.5	2,607,709,506.66	344.11
	Choline	C_5_H_13_NO	0.948	1,702,868,432.32	104.11
	Betaine	C_5_H_11_NO_2_	0.952	815,230,823.19	117.08
	2-(1*H*-indol-3-yl)-3-[4-(trifluoromethyl)phenyl]acrylonitrile	C_18_H_11_F_3_N_2_	10.763	484,746,646.13	312.09
	2-(3,4-dihydroxyphenyl)acetamide	C_8_H_9_NO_3_	0.931	464,700,110.40	167.06
	Isoamylamine	C_5_H_13_N	0.916	204,192,224.93	87.1
	Palmitoleic acid	C_16_H_30_O_2_	15.894	188,602,459.59	254.22
	α-Linolenic acid	C_18_H_30_O_2_	20.046	75,757,786.32	278.44
**MEA**	2,2,6,6-Tetramethyl-1-piperidinol (TEMPO)	C_9_H_19_NO	12.259	312,358,366.23	156.14
	9-Oxo-10(*E*),12(*E*)-octadecadienoic acid	C_18_H_30_O_3_	17.615	128,180,581.48	294.22
	13-hydroperoxy-9*Z*,11*E*-octadecadienoic acid	C_18_H_32_O_4_	17.427	123,110,274.85	312.23
	ethyl 3-oxo-5,6-diphenyl-2,3- dihydropyridazine-4-carboxylate	C_19_H_16_N_2_O_3_	17.351	73,810,505.50	320.12
	Ouabain	C_29_H_44_O_12_	20.518	57,317,714.92	584.66
	(3*S*,3a*R*,4*S*,4a*R*,7a*R*,8*R*,9a*R*)-3,4a,8- trimethyl-2,5-dioxo- 2*H*,3*H*,3a*H*,4*H*,4a*H*,5*H*,7a*H*,8*H*,9*H*,9a*H*azuleno[6,5-*b*]furan-4-yl 2-methylpropanoate	C_19_H_26_O_5_	14.874	51,133,618.50	334.18
**MH**	5-(2-Thienyl)nicotinic acid	C_10_H_7_NO_2_S	0.927	209,833,574.73	205.02
	Oleamide	C_18_H_35_NO	21.564	113,705,808.63	281.27
	4-{[(4,6-Dimethoxypyrimidin-2-yl)amino]methylidene}-2-phenyl-4,5-dihydro-1,3-oxazol-5-one	C_16_H_14_N_4_O_4_	11.582	85,055,308.35	326.1
	1,2-dihydroxyheptadec-16-yn-4-yl acetate	C_19_H_34_O_4_	15.669	79,749,057.14	326.25
	Adenosine	C_10_H_13_N_5_O_4_	1.024	79,366,759.62	267.1
**SE**	Betaine	C_5_H_11_NO_2_	0.902	615,708,699.02	117.08
	dl-β-Leucine	C_6_H_13_NO_2_	0.901	104,452,391.68	131.09
	α-Eleostearic acid	C_18_H_30_O_2_	16.928	82,954,091.16	278.22
	Choline	C_5_H_13_NO	0.906	62,987,796.05	104.11
	Palmitoleic acid	C_16_H_30_O_2_	16.654	37,079,182.87	254.22
	Stearoyl Ethanolamide	C_20_H_41_NO_2_	22.8	33,398,733.18	309.3
	Levalbuterol	C_13_H_21_NO_3_	13.398	25,493,790.28	239.15
**SEA**	Hexadecanamide	C_16_H_33_NO	21.916	353,793,967.91	255.26
	2,2,6,6-Tetramethyl-1-piperidinol (TEMPO)	C_9_H_19_NO	12.294	274,478,961.83	156.14
	Ethyl palmitoleate	C_18_H_34_O_2_	18.681	75,543,295.89	282.26
	2-(3-Chloro-2-fluorophenyl)-2,3-dihydroisothiazol-3-one	C_9_H_5_ClFNOS	0.835	60,193,728.47	228.98
	Cafestol	C_20_H_28_O_3_	17.434	39,577,349.70	316.2
	Shogaol	C_17_H_24_O_3_	13.132	16,983,601.06	276.17
**SH**	Octadec-9-ynoic acid	C_18_H_32_O_2_	17.367	18,964,856.34	280.45
	3,5-di-*tert*-Butyl-4-hydroxybenzaldehyde	C_15_H_22_O_2_	16.951	17,509,512.15	234.16
	Sphingosine (d18:1)	C_18_H_37_NO_2_	20.015	16,138,319.09	299.5
	γ-Linolenic acid ethyl ester	C_20_H_34_O_2_	17.72	13,897,675.48	306.26
	8*Z*,11*Z*,14*Z*-Eicosatrienoic acid	C_20_H_34_O_2_	18.548	8,618,378.15	306.2545

**ME**: Maceration—Ethanol; **MEA**: Maceration—Ethyl Acetate; **MH**: Maceration—*n*-Hexane; **SE**: Soxhletation—Ethanol; **SEA**: Soxhletation—Ethyl Acetate; **SH**: Soxhletation—*n*-Hexane.

**Table 2:** LC_50_ value of *C. lentillifera* in a cytotoxicity test in BUD-8 normal cell lines.

Hours of Incubation	LC_50_ (μg/mL)					
	ME	MEA	MH	SE	SEA	SH
**24 h**	2719.00	4677.87	4397.29	2111.90	4190.67	8445.30
**48 h**	1867	1354	2090	1575	3440	5000

**ME**: Maceration—Ethanol; **MEA**: Maceration—Ethyl Acetate; **MH**: Maceration—*n*-Hexane; **SE**: Soxhletation—Ethanol; **SEA**: Soxhletation—Ethyl Acetate; **SH**: Soxhletation—*n*-Hexane.

**Table 3:** IC_50_ values (μg/mL) exhibited by the *C. lentillifera* on different cancer (colorectal, hepatoma, breast, leukemia, and normal (monocular blood and epithelial) cell lines.

Extract	Colorectal	Hepatoma		Breast Cancer Cell Lines			Leukemia		Control Cell Lines	
	HCT-8	Hep G2	KAIMR C1	MDA-MB-231	MCF-7	KG-1a	K-562	HL-60	Human Epithelial	PBMC
**ME**	170.10	1065.00	160.80	320.50	100.90	508.50	1500.30	315.50	850.90	4040.00
**MEA**	540.60	744.60	885.01	1230.50	1800.30	1260.80	1328.50	2505.90	4040.60	3074.10
**MH**	184.00	283.00	100.50	280.50	350.20	340.50	2705.60	3805.50	5009.00	7080.00
**SE**	160.50	870.60	640.60	104.10	840.60	645.60	954.60	1085.01	2008.00	3024.30
**SEA**	587.50	934.30	1084.00	2085.00	1509.00	2580.50	1934.30	3084.00	4031.10	4598.00
**SH**	2003.50	3282.00	3056.00	3824.80	2506.00	2503.10	3203.00	3003.00	5056.00	5033.00
**M/** **Control**	0.188	0.101	0.798	0.665	1.409	0.167	0.458	1.034	0.134	0.204

**ME**: Maceration—Ethanol; **MEA**: Maceration—Ethyl Acetate; **MH**: Maceration—*n*-Hexane; **SE**: Soxhletation—Ethanol; **SEA**: Soxhletation—Ethyl Acetate; **SH**: Soxhletation—*n*-Hexane.

## Data Availability

The data sets generated and/or analyzed in this study are available in the manuscript or can be requested from the author (F.N.) upon reasonable request.

## Sample Availability

Samples of the compounds are not available from the authors.
